# Chicken oviduct—the target tissue for growth hormone action: effect on cell proliferation and apoptosis and on the gene expression of some oviduct-specific proteins

**DOI:** 10.1007/s00441-014-1860-6

**Published:** 2014-04-18

**Authors:** Anna Hrabia, Agnieszka Leśniak-Walentyn, Andrzej Sechman, Arieh Gertler

**Affiliations:** 1 Department of Animal Physiology and Endocrinology, University of Agriculture in Krakow, Al. Mickiewicza 24/28, 30-059 Krakow, Poland; 2 Robert H. Smith Faculty of Agriculture, Food and Environment, The Hebrew University of Jerusalem, Rehovot, Israel

**Keywords:** Growth hormone, Proliferation, Apoptosis, Ovalbumin, Ovocalyxin, Oviduct, Chicken

## Abstract

The aim of this study was to examine the in vivo effect of growth hormone (GH) on cell proliferation and apoptosis and on the gene expression of selected proteins in the chicken oviduct before sexual maturity (first oviposition). Ten-week-old Hy-Line Brown chickens were injected three times a week with 200 μg · kg^-1^ body weight of recombinant chicken GH (cGH) until 16 weeks of age. Control hens received 0.9 % NaCl with 0.05 % bovine serum albumin as a vehicle. Treatment with cGH increased (*P* < 0.05) oviduct weight at 16 weeks of age, i.e. 1–2 weeks before onset of egg laying. The highest number of proliferating (determined by proliferating cell nuclear antigen [PCNA] immunocytochemistry) and apoptotic (determined by TUNEL assay) cells in the oviduct was found in the mucosal epithelium, and the lowest in the stroma. Administration of cGH did not increase (*P* > 0.05) the number of PCNA-positive cells but it decreased (*P* < 0.01) the number of TUNEL-positive cells, thus increasing the proliferating-to-apoptotic cell ratio in the oviduct. Gene expression (determined by real-time polymerase chain reaction) of apoptosis-related caspase-2 in the magnum and caspase-3 in the magnum and isthmus and their activity (determined by fluorometric assay) in the magnum were attenuated (*P* < 0.05) in cGH-treated hens. The gene expression of the magnum-specific ovalbumin and the shell-gland-specific ovocalyxins 32 and 36 was increased (*P* < 0.05) in cGH-treated chickens. In contrast, the expression of Bcl-2 and of caspases 8 and 9 was not affected by cGH in any of the oviductal segments. The results suggest that GH, via the orchestration of apoptosis and expression of some oviduct-specific proteins, participates in the development and activity of the chicken oviduct prior to the onset of egg laying.

## Introduction

The hen oviduct is of special interest to commercial egg producers because disrupted activity or pathological changes directly affect egg quality and ultimately decrease economic profitability (Chousalkar and Roberts 2008). Moreover, in biotechnological applications, the use of chicken oviduct as a bioreactor for the production of human therapeutic proteins has garnered interest. The avian oviduct is divided morphologically and functionally into five regions. The infundibulum engulfs the ovum, which is released upon ovulation; this is where fertilization takes place and the first layer of egg white is secreted. The magnum produces most (∼15 g) of the egg white. Both inner and outer shell membranes are formed in the isthmus. Calcification of the eggshell and its subsequent pigmentation and cuticle deposition occur in the shell gland. When these processes are complete, expulsion of the egg through the vagina begins.

The avian oviduct undergoes dynamic morphological and functional changes during the reproductive cycle. The most important processes involved in avian oviduct development and function seem to be cell proliferation, differentiation and apoptosis. Apoptosis allows the controlled removal of aged, damaged, infected or unwanted cells, maintaining homeostasis and remodelling in multicellular organisms. The control of cell death is dependent on competing pro- and anti-apoptotic signals (Elmore 2007; Hawkins and Devitt 2013; McIlwain et al. 2013). Among these are proteins from the Bcl-2 family, survivin, an inhibitor of T-cell apoptosis (Ita) and the apoptosis-inducing factor FasL. The Bcl-2 family consists of not only proteins that inhibit apoptosis, but also proteins that stimulate it (Watters and Lavin 1999). For instance, the overexpression of Bcl-2 inhibits apoptosis (Allen et al. 1998). The caspases, i.e. intracellular proteases whose activation leads to the cleavage of numerous cellular proteins, play specific roles in apoptosis. In general, two main apoptotic pathways can be found: extrinsic and intrinsic. Each of these activates its own initiator caspases, such as caspases 1, 2, 8, 9 and 10, which in turn activate executioner caspases (caspases 3, 6 and 7), resulting in cell death. Caspase-3 is considered to be the most important of the executioner caspases and is activated by any of the initiator caspases (Hengartner 2000; Elmore 2007; McIlwain et al. 2013).

The control of oviduct development and activity is mainly orchestrated by ovarian steroids. Oestrogens are the key steroids controlling oviductal growth and activity, via the regulation of cell proliferation and differentiation, and the synthesis of egg proteins (Dougherty and Sanders 2005). Ni et al. (2007) have found the growth hormone (GH) receptor in the shell gland of the chicken oviduct and, recently, Hrabia et al. (2013) have demonstrated the differential mRNA expression and protein localization of GH receptors in the infundibulum, magnum, isthmus and shell gland of laying hens and in the vagina (unpublished data); these findings suggest that chicken oviduct is also a GH-responsive organ. Moreover, hens injected with exogenous GH show increased eggshell quality of the eggs laid near the end of the laying period (Donoghue et al. 1990).

Accumulating evidence in mammals indicates the involvement of GH in the regulation of uterine function. Expression of GH in the human uterus (Sbracia et al. 2004; Slater et al. 2006) and of GH receptor in human, mouse and bovine uterus (Sharara et al. 1994; Kirby et al. 1996; Kölle et al. 2001; Pershing et al. 2002; Sbracia et al. 2004; Rhoads et al. 2008) has been demonstrated. The promotion of uterine growth by GH and the involvement of the mitogenic action of GH in uterine and cervical cancers have also been reported (Slater et al. 2006; Pandey et al. 2008). In addition, GH has been implicated in the regulation of implantation in mice (Zaczek et al. 2002). However, the role of GH in the chicken oviduct is unknown.

Therefore, in the present study, the effect of exogenous chicken GH (cGH) on cell proliferation and apoptosis has been examined in the domestic hen oviduct during sexual maturation. Since numerous genes are involved in these events and are thus potential targets of GH action, we have determined the ability of GH to regulate the expression of anti-apoptotic Bcl-2 protein and the activities of the most important caspases involved in the various apoptotic pathways. As the development of the oviduct is associated with differentiation and specialization of cells of the particular oviductal segments for the production of egg-specific proteins, the possibility that GH regulates the synthesis of these proteins in the chicken oviduct has also been evaluated. The following proteins have been examined: ovalbumin, a protein that is synthesized mainly in the magnum and that constitutes 54 % of the egg white (Nys et al. 2001), and the recently described eggshell-matrix proteins produced predominantly in the shell gland (Gautron et al. 2001, 2007; Hincke et al. 2003), i.e. ovocalyxin-32, which influences the variability of crystal traits and, in turn, the thickness profile of the shell (Takahashi et al. 2010; Dunn et al. 2012; Fulton et al. 2012) and ovocalyxin-36, whose expression is strongly upregulated during shell calcification and which has antimicrobial activity and might play a role in the natural defence of the egg against pathogens (Gautron et al. 2011).

## Materials and methods

### Chemicals

Recombinant cGH was prepared as described by Paczoska-Eliasiewicz et al. (2006) and purchased from Protein Laboratories Rehovot (Rehovot, Israel). The in situ cell-death detection kit (POD–horseradish peroxidase) was from Roche Diagnostics (Mannheim, Germany). The chemicals for immunocytochemistry were obtained from the following companies: mouse monoclonal antibody against proliferating cell nuclear antigen (PCNA) from Novocastra Laboratories (Newcastle upon Tyne, UK) and biotinylated goat antimouse immunoglobulin, normal goat serum and Vectastain ABC kit from Vector Laboratories (Burlingame, USA). The chemicals for reverse transcription plus the polymerase chain reaction (RT-PCR) were purchased from the following companies: TRI-reagent from MRC, Cincinnati (Ohio, USA) and High-Capacity cDNA Reverse Transcription Kit, TaqMan Gene Expression Master Mix, TaqMan Gene Expression Assays and Eukaryotic 18S rRNA Endogenous Control from Applied Biosystems (Foster City, Calif., USA). Caspase fluorometric assay kits were from BioVision (Milpitas, Calif., USA). All other reagents were obtained from ICN Biomedicals (Aurora, Ill., USA), Sigma (St. Louis, Mo., USA) or POCH (Gliwice, Poland).

### Animals and experimental design

Animal experiments were conducted according to research protocols approved by the Local Animal Ethics Committee in Kraków, Poland. Immature Hy-Line brown chickens (layer strain) were purchased at 9 weeks of age from the commercial farm Drobeco (Palowice, Poland). Birds were caged individually under a photoperiod of 14 light:10 dark (lights on at 0700 h and off at 2100 h) with free access to commercial food (11.5 MJ · kg^-1^, 15 % protein) and water. Ten-week-old pullets (*n* = 12) were divided equally into control and experimental groups. Control birds were injected subcutaneously (abdominal area) with 0.9 % (w/v) NaCl containing 0.05 % (w/v) bovine serum albumin (BSA), and the experimental group received recombinant cGH at a dose of 200 μg in 0.1 ml · kg^-1^ body weight diluted in 0.9 % NaCl with 0.05 % BSA. Injections were performed three times a week in the morning. At the age of 16 weeks (about 1–2 weeks before puberty), birds were decapitated and the oviducts were rapidly isolated. After the oviducts had been weighed, four oviductal parts, namely, the infundibulum, magnum, isthmus and shell gland, were collected. Tissue samples were immediately frozen and kept at –80 °C until the determination of caspase activity or were placed into RNA later and stored at –20 °C until total RNA isolation. The other tissue fragments were fixed in freshly prepared 4 % (v/v) buffered formalin, processed and embedded in paraffin wax for subsequent localization of proliferating and apoptotic cells as described previously (Hrabia et al. 2011).

### Evaluation of proliferation by PCNA immunocytochemistry

After deparaffinization in xylene and rehydration in alcohols, 6-μm-thick sections were rinsed in water and treated with 0.5 % (v/v) H_2_O_2_ in methanol to block endogenous peroxidase activity. After being washed in water, slides were heated in a citric buffer (pH 6.0, 75 °C, 20 min) followed by incubation with 5 % (v/v) normal goat serum in TBST buffer (TRIS buffer saline + 0.1 % v/v Tween 20, at room temperature, 10 min). Sections were then incubated for 60 min with mouse monoclonal antibody against PCNA, a marker of the S phase of the cell cycle (dilution 1:150), followed by washes with TBS and incubation with biotinylated goat antimouse antibody (35 min, dilution 1:300) and with Vectastain ABC kit (30 min). The colour reaction was developed by incubation with diaminobenzidine (DAB) and H_2_O_2_ solution. Control slides were prepared by using TBST instead of the primary antibody. Slides were examined under a light microscope (Zeiss Jena, Germany). Proliferating (PCNA-positive) cells were counted with a computerized image-analysis system (MultiScanBase v. 14.02, Computer Scanning System, Warsaw, Poland) in 10 random areas (50 × 50 μm) of each examined magnum, isthmus and shell gland and averaged for each bird. The mean value was calculated from six chickens. The infundibulum was omitted in the cell-number calculation as it was not possible accurately to measure the area of mucosa.

### Apoptosis evaluation by TUNEL assay

For the evaluation of apoptosis, deparaffinized and rehydrated 6-μm-thick sections of oviduct tissue were incubated with proteinase K (20 μg · ml^-1^) in 10 mM TRIS-HCl, pH 7.4, at 37 °C for 20 min and apoptotic cells were detected by the terminal deoxynucleotidyl transferase-mediated dUTP nick-end labelling (TUNEL) method by using the in situ cell-death detection kit POD according to the protocol provided by the manufacturer (Roche). Negative controls were prepared by incubating slides without terminal deoxynucleotidyl transferase. To visualize the immunoreaction products, sections were incubated with a mixture of DAB and H_2_O_2_. Slides were examined and TUNEL-positive cells were counted as for the PCNA-positive cells.

### RNA isolation, cDNA synthesis and quantitative PCR

Total RNA was extracted from the tissues by using TRI-reagent according to manufacturer’s recommendations (MRC). RNA (2 μg from each tissue) was reverse-transcribed with a High-Capacity cDNA Reverse Transcription Kit including random primers. Samples were incubated in a thermocycler (Mastercycler Gradient; Eppendorf, Hamburg, Germany) according to the following thermal profile: 25 °C for 10 min, 37 °C for 120 min and 85 °C for 5 min. The obtained cDNA was used in real-time PCR for Bcl-2, caspases 2, 3, 8 and 9, ovalbumin and ovocalyxins 32 and 36 in a 96-well thermocycler (StepOne Plus; Applied Biosystems, USA) according to the recommended cycling program: 2 min at 50 °C, 10 min at 95 °C, 40 cycles of 15 s at 95 °C and 60 s at 60 °C. The multiplex real-time quantitative PCRs (qPCRs) for the examined genes were performed in a 10 μl volume containing 5 μl TaqMan Gene Expression Master Mix, 0.5 μl TaqMan Gene Expression Assays with specific TaqMan MGB-probe and one pair of primers designed by Applied Biosystems, 0.5 μl Eucaryotic 18S rRNA Endogenous Control (pair of primers and TaqMan probe-labelled VIC/TAMRA as a reference gene), 3 μl water and 1 μl cDNA (ten-times-diluted sample after the RT step). The TaqMan Gene Expression Assay parameters are shown in Table 1. Each sample was run in duplicate. A no-template control was included in each run. Relative quantification of the investigated genes was calculated after normalization with the 18S rRNA transcript and by employing expression in the infundibulum of the control group as the calibrator for Bcl-2 and caspases by using the 2^-ΔΔCt^ method. In the case of ovalbumin determined in the magnum and ovocalyxins examined in the shell gland, the control group was used as the calibrator. Quantification was performed by using StepOne integrated software.

**Table 1:** TaqMan probe sequences and size of amplicons generated by real-time polymerase chain reaction assay for chicken Bcl-2, caspases 2, 3, 8 and 9, ovalbumin and ovocalyxins 32 and 36 in the chicken oviduct

Gene	Genbank accession number	TaqMan probe (FAM5′ → 3′NFQ)	Amplicon (bp)
Bcl-2 protein	NM_205339.1	AAAGGCATCCCATCCTC	72
Caspase-2	NM_001167701.1	AAGCCTCCTGCAACTGT	97
Caspase-3	AF083029.1	CTGCTCCAGGCTACTACT	72
Caspase-8	NM_204592.2	CCGGCATTGTAGTTTC	88
Caspase-9	XM_424580.3	CAAAGCTCAGGAAATTG	51
Ovalbumin	NM_205152.2	TCTGTCTGGCATCTCC	85
Ovocalyxin-32	AB547158.1	CCCGTGTGCTCTGTTG	79
Ovocalyxin-36	NM_001030861.1	CCCCAGAGAAAGCTTC	77

### Caspase-activity assays

The enzymatic activity of caspases in the oviductal parts of control and cGH-treated chickens was measured according to Monroe et al. (2002) with ready-to-use fluorometric assay kits. Briefly, tissue samples were homogenized in lysis buffer (100 mg tissue per 300 μl buffer) and centrifuged. Supernatants were collected and total protein concentrations were determined by the Bradford method. A 100 μg aliquot of protein from each sample or blank control was transferred in duplicate to 96-well microplates. Immediately after a 2 h incubation at 37 °C in reaction buffer with substrate, fluorescence at 400 nm excitation and 505 nm emission was read in a Fluorescence Microplate Reader FLx800 (BioTek Instruments, Winooski, Vt., USA). The activity of caspases 2, 3, 8 and 9 in the samples was normalized and expressed as the percentage activity relative to the infundibulum of the control group (set at 100 %).

### Statistical analysis

For comparison of the means of the control and cGH-treated groups, the Student *t*-test or Mann–Whitney Rank Sum test was used. Differences were considered significant at *P* < 0.05. Calculations were performed with Sigma Stat 2.03 (Systat Software). Data are presented as means ± SEM.

## Results

**Fig. 1:**
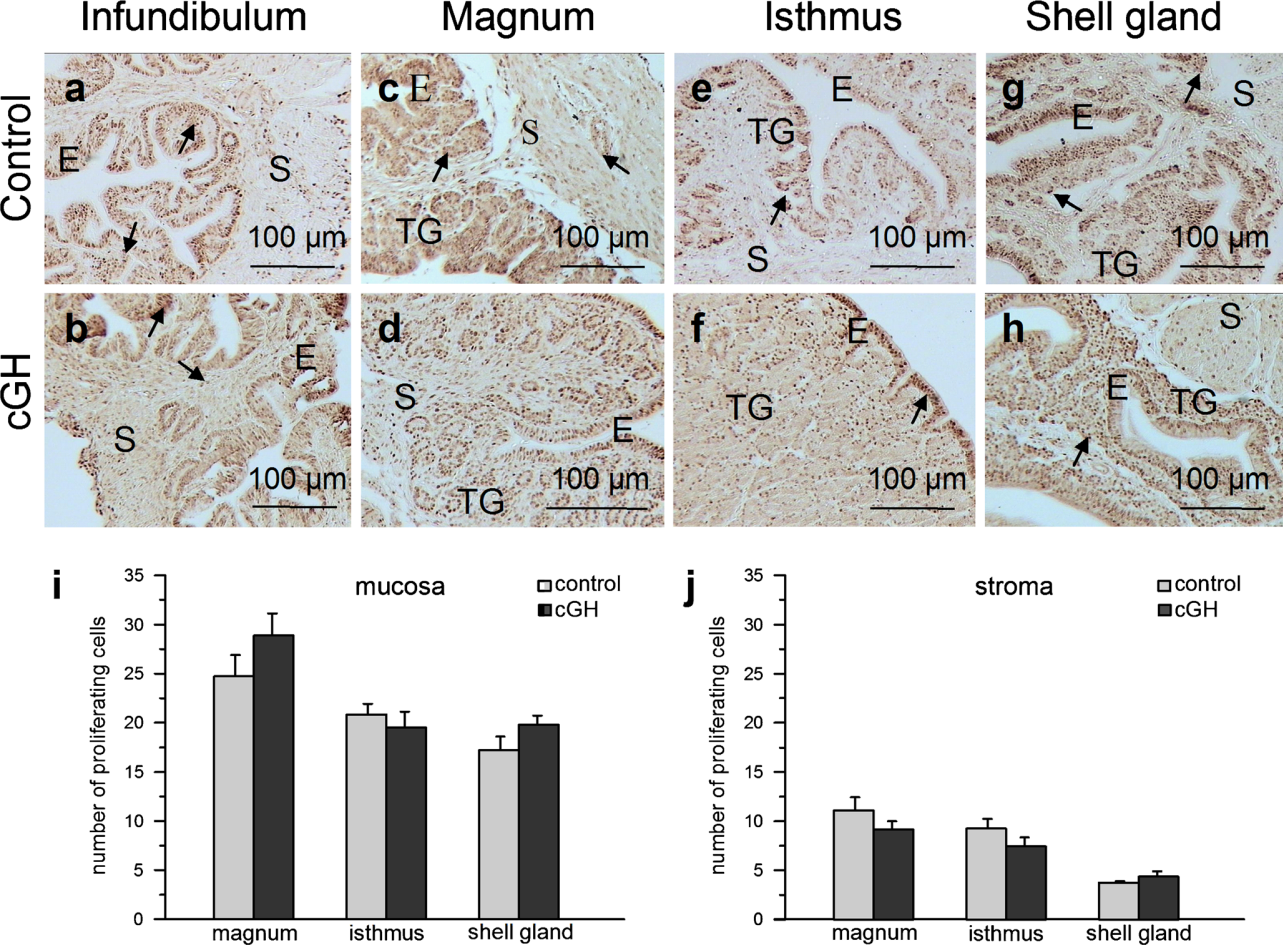
Localization and number of proliferating (PCNA-positive) cells in the chicken oviduct after chicken growth hormone (*cGH*) administration before maturation (representative of 6 birds). **a**, **c**, **e**, **g** Localization of proliferating cells (*arrows*) in the infundibulum, magnum, isthmus and shell gland, respectively, of the control group. **b**, **d**, **f**, **h** Localization of proliferating cells in the infundibulum, magnum, isthmus and shell gland, respectively, of the cGH-treated birds. Numerous proliferating cells are present in the mucosal epithelium (*E*) and tubular glands (*TG*). A lower abundance of proliferating cells are localized in the stroma (*S*; connective tissue + muscles). Note that proliferating cells occur at a similar frequency in the oviductal segments of control and cGH-treated chickens. **i**, **j** Number of proliferating cells in the mucosa (epithelium + tubular glands) and stroma, respectively, of the magnum, isthmus and shell gland in control and cGH-treated groups. Each value represents the mean ± SEM. PCNA-positive cells counted on 10 randomly chosen fields (50 μm x 50 μm) of each magnum, isthmus and shell gland were averaged for each bird and for that microscopic field size and subsequently the mean value for six chickens was calculated

**Fig. 2:**
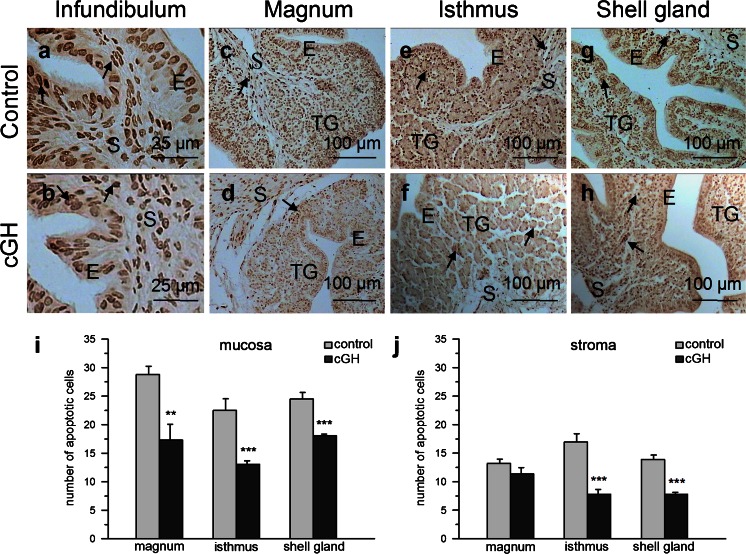
Localization and number of apoptotic (TUNEL-positive) cells in the chicken oviduct after cGH administration before maturation (representative of 6 birds). **a**, **c**, **e**, **g** Localization of apoptotic cells (*arrows*) in the infundibulum, magnum, isthmus and shell gland, respectively, of the control group. **b**, **d**, **f**, **h** Localization of apoptotic cells in the infundibulum, magnum, isthmus and shell gland, respectively, of the cGH-treated birds. Many apoptotic cells are observed in the mucosal epithelium (*E*) and tubular glands (*TG*). Fewer apoptotic cells are localized in the stroma (*S*; connective tissue + muscles). Note that TUNEL-positive cells are less abundant in the cGH-treated group than in the control group. **i**, **j** Number of apoptotic cells in the mucosa (epithelium + tubular glands) and stroma, respectively, of the magnum, isthmus and shell gland in control and cGH-treated groups. Each value represents the mean ± SEM. **P < 0.01, ***P < 0.001 compared with control. TUNEL-positive cells counted on 10 randomly chosen fields (50 μm × 50 μm) of each magnum, isthmus and shell gland were averaged for each bird and for that microscopic field size and subsequently the mean value for six chickens was calculated

The effects of cGH on body and oviduct weights are summarized in Table 2. cGH had no effect on body weight, whereas an increase (*P* < 0.05) in oviduct weight and oviduct index (oviduct weight/body weight × 100) was observed. Immunocytochemical analysis of proliferating cells showed their presence in all examined segments of the oviduct in control and cGH-treated groups (Fig. 1a–h). Within the oviductal wall, the largest number of PCNA-positive cells was observed in the mucosal epithelium; such cells were less abundant in the mucosal tubular glands (note that, in the infundibulum, the tubular glands were not developed) and only a few were found in the stroma (muscle + connective tissue). The numbers of proliferating cells per unit area (50 μm × 50 μm) in the oviducts of control and experimental groups are presented in Fig. 1i, j and follow the order magnum ≥ isthmus > shell gland for both the oviduct mucosa and the stroma. GH treatment had no effect on proliferating cell number. TUNEL-positive apoptotic cells were detected in the oviduct parts of both examined groups (Fig. 2a–h). Within the oviduct wall, the highest number of apoptotic cells was found in the mucosal epithelium; such cells were less abundant in the mucosal tubular glands and least abundant in the stroma. The number of apoptotic cells per unit area (50 μm × 50 μm) in the mucosa and stroma of the control group did not differ (*P* > 0.05) among oviductal parts. Administration of cGH decreased the number of apoptotic cells in the mucosa of the magnum (*P* < 0.01), isthmus (*P* < 0.001) and shell gland (*P* < 0.001), by 40 %, 42 % and 26.5 %, respectively, and in the stroma of the isthmus (*P* < 0.001) and shell gland (*P* < 0.001) by 54 % and 44 %, respectively (Fig. 2i, j).

**Table 2:** Effect of chicken growth hormone (*cGH*) administration on body weight, oviduct weight and oviduct index (means ± SEM from six birds) in growing chickens; *P < 0.05, different from control

Measured parameter	Control^a^	cGH^a^
Initial body weight (g) at age 10 weeks	876 ± 26.3	866 ± 23.8
Final body weight (g) at age 16 weeks	1502 ± 22.5	1517 ± 19.6
Oviduct weight (g)	6.34 ± 1.54	18.65 ± 6.86*
Oviduct index (%)	0.43 ± 0.11	1.22 ± 0.27*

^a^Treatments (three times a week) from 10 to 16 weeks of age

**Fig. 3:**
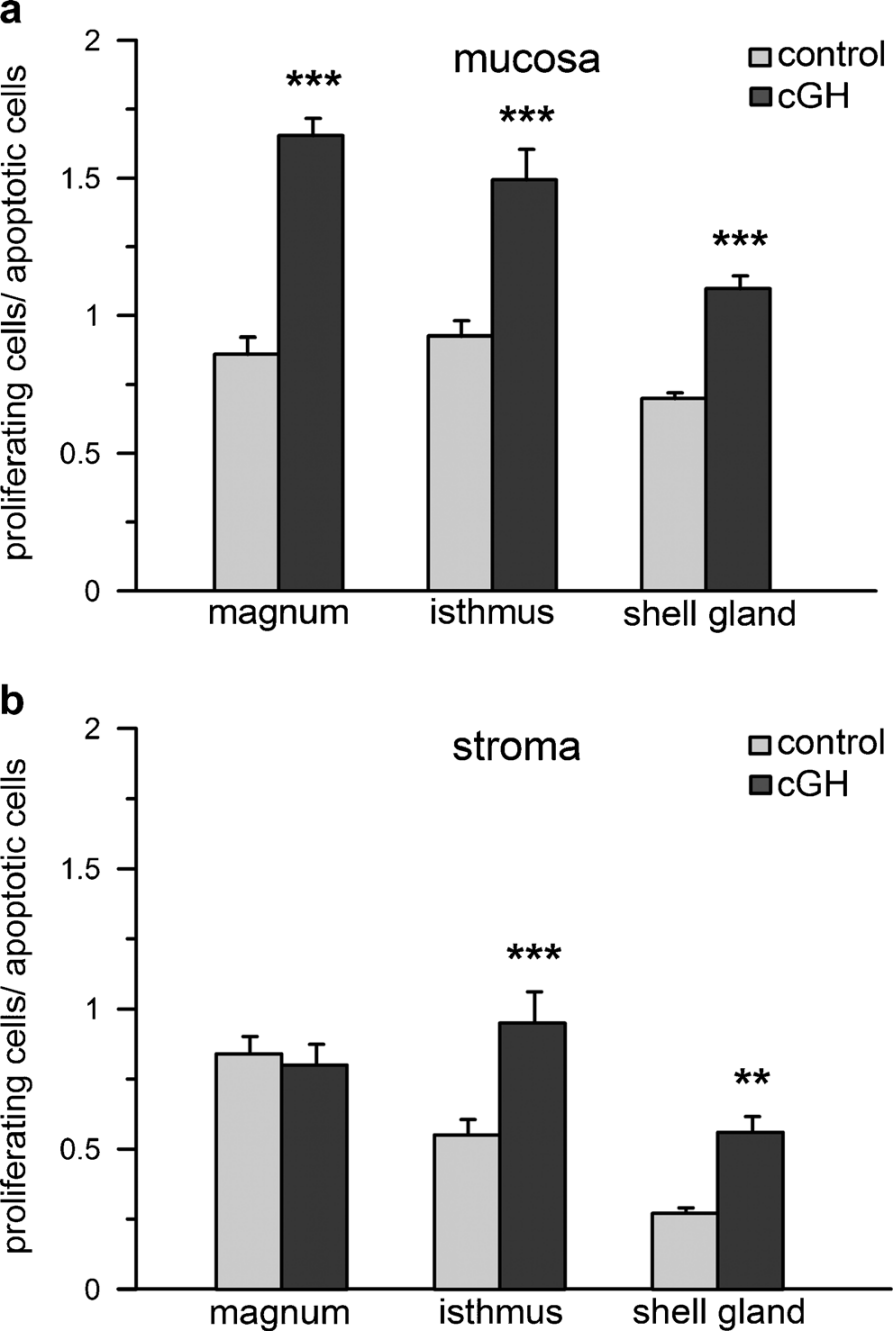
Ratio of proliferating-to-apoptotic cells in the mucosa (**a**) and stroma (**b**) of control and cGH-treated chickens before maturation. Each value represents the mean ± SEM. **P < 0.01, ***P < 0.001 compared with control

The proliferation-to-apoptosis ratio in the mucosa of the magnum, isthmus and shell gland was higher (*P* < 0.001) by 93 %, 61 % and 52 %, respectively, in cGH-treated chickens (Fig. 3a), whereas in the stroma, this ratio was elevated by 73 % and 107 % in the isthmus (*P* < 0.001) and shell gland (*P* < 0.01), respectively, after cGH injections (Fig. 3b).

**Fig. 4:**
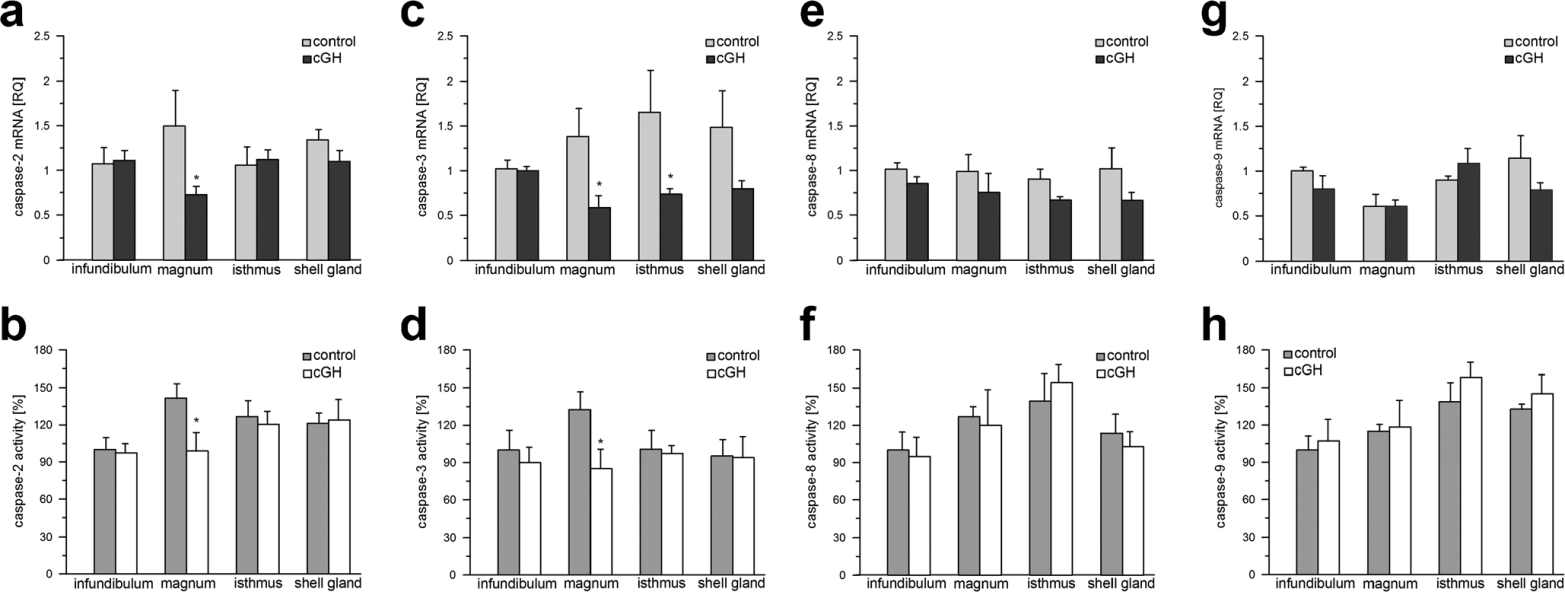
Effect of cGH on caspase-2 (**a**, **b**), caspase-3 (**c**, **d**), caspase-8 (**e**, **f**) and caspase-9 (**g**, **h**) gene expression and activity, respectively, in the chicken oviduct before maturation. Each value in **a**, **c**, **e** and **g** represents the mean of relative quantity (*RQ*) ± SEM from six chickens standardized to control expression in the infundibulum. Each value in **b**, **d**, **f** and **h** represents the mean of relative activity (%) ± SEM from six chickens standardized to control activity, which was set at 100 %, in the infundibulum. *P < 0.05 compared with control

The proliferation-related gene *Bcl-2* showed the same level of expression in all oviductal regions and was not changed by cGH administration (data not shown). The mRNA expression and activity of the apoptosis-related genes for caspases 2, 3, 8 and 9 in the infundibulum, magnum, isthmus and shell gland of control and cGH-treated chickens are presented in Fig. 4a–h. Lower (*P* < 0.05) mRNA expression was found for caspase-2 in the magnum (by 51 %) and for caspase-3 in the magnum (by 58 %) and isthmus (by 55 %) of cGH-treated hens (Fig. 4a, c). Administration of cGH caused a decrease (*P* < 0.05) in caspase-2 activity (by 30 %) and caspase-3 activity (by 36 %) in the magnum (Fig. 4b, d).

**Fig. 5:**
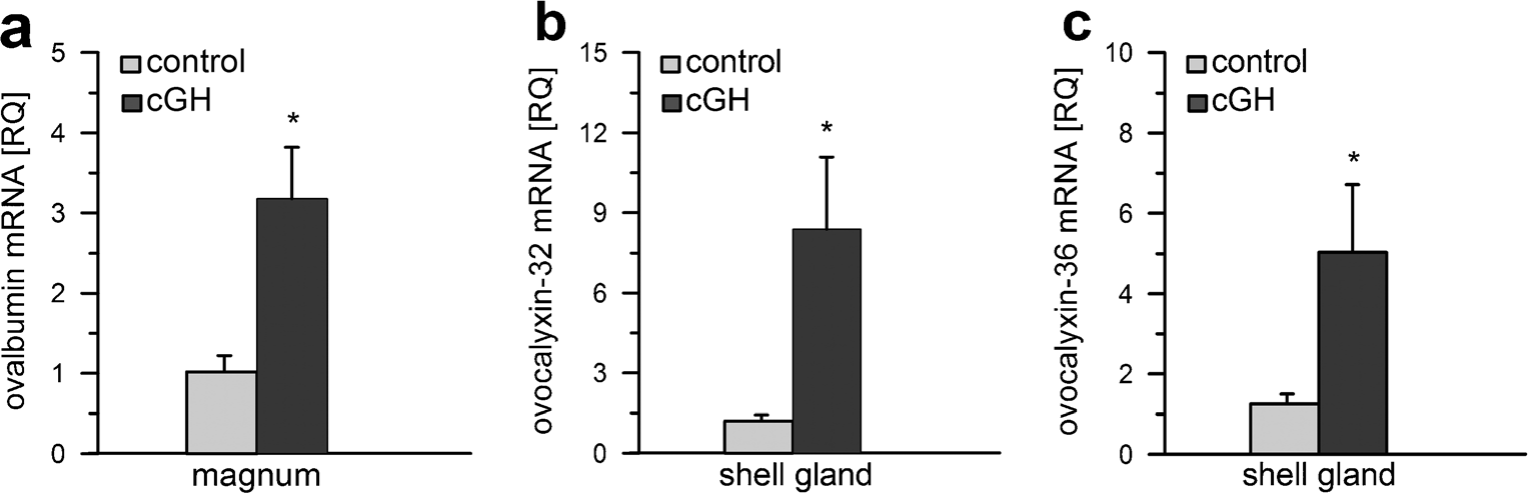
Effect of cGH on gene expression of ovalbumin (**a**) in the magnum and ovocalyxin-32 (**b**) and ovocalyxin-36 (**c**) in the shell gland of chickens before maturation. Each value represents the mean of relative quantity (*RQ*) ± SEM from six chickens standardized to control expression. *P < 0.05 compared with control

The effect of cGH administration on selected oviduct-specific gene expression is shown in Fig. 5. cGH stimulated (*P* < 0.05) ovalbumin mRNA expression in the magnum by 211 % and ovocalyxins 32 and 36 in the shell gland by 592 % and 303 %, respectively.

## Discussion

The present study demonstrates, for the first time, the effect of recombinant cGH on chicken oviduct development and function. First, the effect of cGH administration on cell proliferation and apoptosis was examined in immature chickens. Both proliferating (PCNA-positive) and apoptotic (TUNEL-positive) cells were localized most frequently to the mucosa and were less abundant in the stroma of the infundibulum, magnum, isthmus and shell gland. This indicates that oviduct growth and specialization before puberty are related to the mucosa, which shows the most dynamic development. The similar localization and frequency of proliferating and apoptotic cells clearly occurs because, in dynamically proliferating tissues, cells that are damaged or incorrect in some way must be eliminated, usually by apoptotic pathways (Elmore 2007). No marked changes were observed in the number of proliferating cells after cGH administration. In contrast, cGH significantly decreased the number of apoptotic cells in the mucosa and stroma of the oviductal parts examined 1–2 weeks before predicted maturation. Thus, GH evidently has an anti-apoptotic effect in the chicken oviduct before puberty, as previously established in the chicken ovary (Hrabia et al. 2011) and in a wide range of mammalian reproductive (Sirotkin and Makarevich 1999; Kölle et al. 2003; Sirotkin 2005; Pandey et al. 2008) and nonreproductive (for reviews, see Sanders and Harvey 2008; Harvey 2010) tissue, including immunological (Jeay et al. 2002; Luna et al. 2013), neural (Shin et al. 2004; Alba-Betancourt et al. 2013) and retinal (Sanders et al. 2005, 2009, 2011) tissues. Although an effect of cGH on cell proliferation has not been detected in the oviduct, cGH might act as a survival factor, since such an effect has been observed in vitro in several avian cells, e.g. the B-cells of the bursa of Fabricius (Rodríguez-Méndez et al. 2010) and retinal (Sanders et al. 2006) and cerebellar (Alba-Betancourt et al. 2013) neurons.

As the balance between cell proliferation and apoptosis is important for the proper development of various tissues, the proliferation-to-apoptosis ratio has been determined in the mucosa and stroma of the oviductal segments. Notably, although a clear effect of cGH on proliferating cell number has not been found in the current investigation, the ratio of proliferating-to-apoptotic cells in the oviductal wall is raised by 52 % to 107 %; thus, cGH shifts the predominance of apoptosis over proliferation and, as a consequence, significantly increases (by 294 %) the weight of the oviduct, whereas the body weight is unchanged. A stronger action of GH has been observed in the mucosa than in the stroma suggesting that the mucosa, in which proliferation is crucial for oviduct growth during maturation (Yu and Marquardt 1973), is mostly susceptible to GH. This justifies the previous suggestion of Hrabia et al. (2013) that GH plays a regulatory role in the activity of the oviduct mucosa, since GH receptors in oviductal wall are localized predominantly in this tissue.

Previous studies have shown an anti-apoptotic effect of GH overexpression, attributable at least in part to a decrease in the expression of Bax, BAD and caspases 3, 8 and 9 and to an increase in Bcl-2 production (Mitsunaka et al. 2001; Jeay et al. 2002; Arnold and Weigent 2004). To clarify the apoptotic targets of GH in the chicken oviductal regions, the mRNA expression of Bcl-2, the initiator caspases 2, 8 and 9 and the executioner caspase-3 has been determined in the oviductal segments after cGH administration. Bcl-2 mRNA expression is not affected by cGH, as observed in chicken embryonic neural retina (Harvey et al. 2006). Similarly, cGH does not change the expression of the mRNA for survivin (another proliferation-enhancing and anti-apoptotic protein) in the hen oviduct (own unpublished data). Among the examined caspases, the expression of caspase-2 mRNA in the magnum and caspase-3 mRNA in the magnum and isthmus is reduced by almost half in cGH-treated hens. The lack of an effect of cGH on the mRNA expression of Bcl-2 and initiator caspases 8 and 9 in this work, as compared with the results of earlier studies (Jeay et al. 2002; Shin et al. 2004; Alba-Betancourt et al. 2013), might be attributable to the experimental conditions, namely the dose of cGH, the frequency of its administration, the type of tissue or the physiological microenvironment of the oviductal tissues. On the other hand, the specific lowering of caspase-3 mRNA expression by cGH in the magnum and isthmus indicates that the executioner caspase-3 is a candidate gene target for GH regulation. This finding extends that of Harvey et al. (2006) who have shown that exogenous GH decreases the expression of caspase-3 in the neural retina.

Since active caspases are required for cell apoptosis, the activities of selected caspases (caspases 2, 8, 9 and 3) have also been measured. We have found that, in the magnum of cGH-treated chickens, caspase-2 and caspase-3 activity decreases. These data are in agreement with previous in vivo and in vitro reports on birds in which GH reduced the activity of caspase-3 in the retina (Harvey et al. 2006; Sanders et al. 2006), bursa of Fabricius (Luna et al. 2013) and cerebellum (Alba-Betancourt et al. 2013). The present data provide additional evidence for the possible role of exogenous GH as an anti-apoptotic factor in the chicken oviduct. The effect of GH on caspase expression and activity, observed mainly in the magnum, suggests that the mechanism of action of GH is cell- and tissue-specific. Moreover, the activity of caspases is regulated by numerous molecules; hence, in the current study, the observed inhibitory effect of cGH on caspase mRNA expression, which is not reflected in the attenuation of caspase activity in the isthmus and shell gland, indicates that additional factors are involved in the activation of the examined caspases. Differential mechanisms of caspase activation in particular sections of the oviduct require additional evaluation.

The most interesting finding in this study is the remarkable stimulation of ovalbumin and ovocalyxin mRNA expression in the magnum and shell gland, respectively, by cGH. This strongly indicates the participation of GH in the control of chicken oviduct secretory activity and supports the proposal of Donoghue et al. (1990) that GH is involved in eggshell formation. The mentioned authors have also observed the increased shell thickness of eggs laid by hens injected with exogenous GH near the end of the egg-laying period. Unfortunately, no ovocalyxin 32 or 36 antibodies are available, making it impossible to examine ovocalyxins at the protein level. However, our current data provide new insights for the development of strategies to improve eggshell characteristics, since ovocalyxin-32 is a candidate marker for eggshell traits in the development of such strategies in commercial layer populations (Takahashi et al. 2010).

The wide range of the physiological effects of GH, including metabolism regulation, immune response, cell migration, proliferation, prevention of cell death and gene transcription (Pilecka et al. 2007; Sirotkin 2005; Harvey 2010), are the results of its direct action via GH receptors localized in target cells or of its indirect action via insulin-like growth factors (IGFs). Thus, some actions of GH in chicken oviduct might be mediated at least in part by IGFs. This suggestion is supported by the finding that, in adult female chickens, exogenous GH treatment markedly increases plasma concentrations of IGF-I and IGF-binding proteins (Scanes et al. 1999). In primary cultures of quail oviduct cells, an elevation of ovalbumin synthesis by IGF-1 in cooperation with oestrogen has been reported (Kida et al. 1995). GH might also induce oestrogen receptors and, in this way, regulate chicken oviduct functions. Of pertinence, a study has demonstrated the direct action of GH on the rat liver leading to an increase oestrogen receptor expression (Freyschuss et al. 1994). Furthermore, the involvement of GH in the regulation of oestrogen sensitivity of the vitellogenic response in the liver of male and female turtles has also been revealed (Ho et al. 1982). Moreover, GH might also exert its effect in the chicken oviduct through autocrine and/or paracrine mechanisms, as has been strongly suggested in other chicken reproductive tissues, i.e. in the ovary (Hrabia et al. 2008, 2011, 2012; Ahumada-Solórzano et al. 2012) and testis (Harvey et al. 2004; Luna et al. 2004; Martínez-Moreno et al. 2011) in which GH is locally produced and exerts several physiological effects. Although no information is yet available as to whether GH is synthesized in the avian oviduct, this possibility is highly likely.

In conclusion, taking into consideration the present results, we suggest that GH, via the orchestration of apoptosis and the expression of some oviduct-specific proteins, participates in the development and activity of the chicken oviduct prior to the onset of egg laying.
